# Active Infective Native and Prosthetic Valve Endocarditis: Short- and Long-Term Outcomes of Patients after Surgical Treatment

**DOI:** 10.3390/jcm10091868

**Published:** 2021-04-26

**Authors:** Mohamed Salem, Christine Friedrich, Mohammed Saad, Derk Frank, Mostafa Salem, Thomas Puehler, Jan Schoettler, Felix Schoeneich, Jochen Cremer, Assad Haneya

**Affiliations:** 1 Department of Cardiovascular Surgery, Christian-Albrechts-University of Kiel, School of Medicine, Arnold-Heller-Str. 3, D-24105 Kiel, Germany; Christine.Friedrich@uksh.de (C.F.); thomas.puehler@uksh.de (T.P.); Jan.Schoettler@uksh.de (J.S.); Felix.schoeneich@uksh.de (F.S.); Jochen.Cremer@uksh.de (J.C.); Assad.Haneya@uksh.de (A.H.); 2 Department of Cardiology and Angiology, Christian-Albrechts-University of Kiel, School of Medicine, Arnold-Heller-Str. 3, D-24105 Kiel, Germany; Mohammed.saad@uksh.de (M.S.); derk.frank@uksh.de (D.F.); Mostafa.Salem@uksh.de (M.S.)

**Keywords:** infective native valve endocarditis, infective prosthetic valve endocarditis, intensive care therapy for infective endocarditis

## Abstract

Background: Active infective endocarditis (IE) is a serious disease associated with high mortality. The current study represents our experience over 18 years with surgical treatment for active infective native and prosthetic valve endocarditis (INVE, IPVE). Method: Analysis of 413 patients (171 with IPVE vs. 242 with INVE) who underwent cardiac surgery due to IE between 2002 and 2020. Results: Patients with IPVE were significantly older (64.9 ± 13.2 years vs. 58.3 ± 15.5 years; *p* < 0.001) with higher EuroSCORE II (21.2 (12.7; 41.8) vs. 6.9 (3.0; 17.0); *p* < 0.001)) and coronary heart disease (50.6% vs. 38.0%; *p* < 0.011). Preoperative embolization was significantly higher within INVE (35.5% vs. 16.4%; *p* < 0.001) with high incidence of cerebral embolization (18.6% vs. 7.6%; *p* = 0.001) and underwent emergency curative surgery than the IPVE group (19.6% vs. 10.6%; *p* < 0.001). However, patients with IPVE were significantly represented with intracardiac abscess (44.4% vs.15.7%; *p* < 0.001). Intraoperatively, the duration of surgery was expectedly significantly higher in the IPVE group (356 min vs. 244 min.; *p* = 0.001) as well as transfusion of blood (4 units (0–27) vs. 2 units (0–14); *p* < 0.001). Post-operatively, the incidence of bleeding was markedly higher within the IPVE group (700 mL (438; 1163) vs. 500 mL (250; 1075); *p* = 0.005). IPVE required significantly more permanent pacemakers (17.6% vs. 7.5%: *p* = 0.002). The 30-day mortality was higher in the IPVE group (24.6% vs. 13.2%; *p* < 0.003). Conclusion: Patients with INVE suffered from a higher incidence of cerebral embolization and neurological deficits than patients with IPVE. Surgical treatment in INVE is performed mostly as an emergency indication. However, patients with IPVE were represented commonly with intracardiac abscess, and had a higher indication of pacemaker implantation. The short- and long-term mortality rate among those patients was still high.

## 1. Introduction

Active infective endocarditis (IE) is a serious disease. Despite improvement in its management, it is still associated with significant morbidity and mortality [1]. The risk factors are multifactorial. The most common causes are due to intravenous drug injections, prosthetic valve implantations and implantable pacemaker devices, patients with chronic indwelling catheters, and immunocompromised patients [2]. Surgical treatment of IE is required in 25–30% in acute cases and 20–40% in subacute and chronic cases [3]. Those surgical indications include recurrent embolism, the presence of prosthetic implantation, progressive heart failure, and the presence of resistant organisms [4,5]. While antibiotic therapy represents the first line in the treatment of IE, surgical treatment remains in many cases a lifesaving and emergency indication. The risk is increased in patients with infective prosthetic valve endocarditis (IPVE) compared to infective native valve endocarditis (INVE). Prior studies show diverging results regarding mortality after operations for IPVE [5,6,7]. This current study aimed to represent our experience over 18 years with surgical treatment in patients with IE and to compare short- and long-term survival after surgical therapy for INVE and IPVE.

## 2. Materials and Methods

### 2.1. Patient Population

The retrospective study included 413 consecutive patients who underwent open cardiac surgery due to IE between January 2002 and February 2020 in our tertiary university hospital. Patients underwent surgery either due to native or prosthetic valve IE exclusively; 171 (41%) patients underwent surgery due to IPVE, while 242 (59%) due to INVE. The primary endpoint was 30-day mortality. Secondary endpoints were long-term survival, intraoperative variables, and post-operative outcomes such as redo-surgery, blood loss, ventilation time, acute renal failure, and neurologic complications. Active IE was defined as patients receiving ongoing antibiotic therapy. The mortality included all causes leading to death during the hospital stay after the surgical treatment of IE within the post-operative 30 days. Data were supplied from the institution’s database and medical records. Follow-up in terms of survival was determined by inquiries at the residents’ registration offices. The study protocol was approved by the local Ethics Committee and informed consent was taken from the patients.

### 2.2. Patient Management

All cases of IE were discussed in our Endocarditis Team, consisting of a cardiologist, cardiac surgeon, and infectious disease consultant. The diagnosis was settled according to the modified Duke Criteria. Patients without microbiological prove of endocarditis was diagnosed according to another major and minor criterion of modified Duke criteria. A transthoracic echocardiogram was performed in all patients without exclusion. The measurements of interest included size and location of vegetation, presence of abscess or valve destruction, and left ventricle ejection fraction (LVEF). The antibiotics treatment starts as soon as IE was diagnosed and an intravenous treatment regime was introduced for at least 4–6 weeks independent of the time of surgery. Blood culture was taken in all patients to identify the organisms according to species and sensitivities. All patients referred with stroke underwent a computer tomography scan of the brain to exclude any risk of bleeding before surgery and to estimate the prognosis if patients have been intubated and in a coma. The neurological status of the patients was evaluated by a consultant neurologist. Perioperative characteristics and clinical variables, risk factors, intraoperative data, and predictors for mortality were analyzed.

### 2.3. Surgical Procedure

All patients underwent curative surgery performed by senior surgeons. The cardiopulmonary bypass (CPB) was performed with direct cannulation of the ascending aorta. Venous drainage was performed either through direct cannulation of the right atrium in cases of aortic valve endocarditis or through double cannulation of superior and inferior vena cava in cases of mitral or tricuspid valve endocarditis followed by cross-clamping of ascending aorta. A choice between biological, mechanical prosthesis, or valve repair was done preoperatively according to the age of the patients, the patient’s preference, and their compliance with long-term anticoagulation, as well as the intraoperative findings and the degree of macroscopic valve destruction. The affected areas underwent extensive debridement with copious irrigation. The defected areas were repaired either through isolated valve replacement or aortic root reconstruction with or without autologous or bovine pericardium in cases of extensive destruction or the presence of an abscess. Continuous CO_2_ insufflation was used as a standard for cardiac de-airing. Transesophageal echocardiography is used for assessment after surgical repair and to control the presence of residual air in the left side of the heart during rewarming.

### 2.4. Statistical Analysis

Statistical analysis was performed using the SPSS Statistics software (Version 24.0, Chicago, IL, USA). The normality of continuous variables was assessed by the Kolmogorov–Smirnov–Test. Normally distributed data are presented as the mean ± standard deviation and not normally distributed data as median with range or interquartile range as appropriate. Categorical variables are displayed as frequency distributions (n) and simple percentages (%). Univariate comparison between the groups for categorical variables was made using the x^2^ and the Fisher’s exact test as appropriate, while quantitative variables were compared by the *t*-test or Mann–Whitney-U-Test if they were not normally distributed. Statistical significance was considered when *p* ≤ 0.05. Variables associated with 30-day mortality were selected due to clinical relevance and included in multivariable logistic regression analysis with backward elimination to determine their relative impact (adjusted odds ratio, OR) on 30-day mortality. Included variables were age, Euro-SCORE II, female gender, coronary heart disease, poor LVEF, presence of previous cardiac surgery, cardiogenic shock, preoperative hemodialysis, preoperative stroke, and presence of an abscess.

## 3. Results

**Figure 1:**
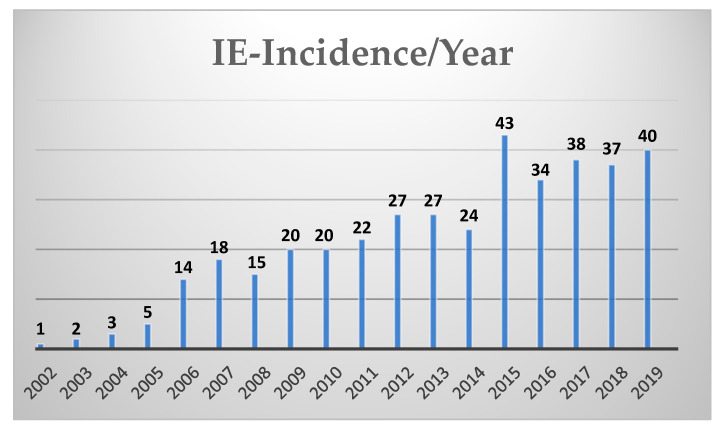
Showing the yearly incidence of infective endocarditis treated surgically over the last 18 years.

Analysis of the demographic and preoperative status showed that INVE present in 242 patients (59%) without history of previous cardiac surgery while 171 patients (41%) underwent surgery due to IPVE. Patient with IPVE were significantly older than patients with INVE (64.9 ± 13.2 years vs. 58.3 ± 15.5 years; *p* < 0.001) with higher percentage of patients older than 70 years (77 (45.0%) years vs. 60 (24.8%) years; *p* < 0.001)). EuroSCORE II was significantly higher among IPVE group (21.2 (12.7; 41.8) vs. 6.9 (3.0; 17.0); *p* < 0.001)). Those patients suffered more significantly from coronary heart disease (50.6% vs. 38.0%; *p* < 0.011), poorer LVEF under 30% (24 (14.9%) vs. 17 (7.4%) years; *p* = 0.017)) and arterial hypertension (1 29 (75.4%) vs. 111 (45.9%); *p* < 0.001). The incidence of infective endocarditis that required surgical intervention increased yearly. Figure 1 represents the course of IE over the last 18 years. 

Intravenous drug abuse was noticed in 23 patients (5.6% of the study population). It was more common in patient with INVE (18 (7.4%) vs. 5 (2.9%); *p* = 0.049). Preoperative embolization was significantly higher among those patients with INVE (35.5% vs. 16.4%; *p* < 0.001). Cerebral embolization was with the highest incidence and represent 58 patients (14%) of the total (18.6% in INVE vs. 7.6% in IPVE; *p* = 0.001). Spleen embolization was the second most common embolization with 17 patients (4.1%). On the other side, patients with IPVE were significantly represented with intracardiac abscess (44.4% vs.15.7%; *p* < 0.001). A history of recurrent endocarditis reaches 14.5% of the total population with a significantly higher incidence in patients with IPVE (43 (25.1%) vs. 17 (7.0%); *p* < 0.001). Among the total study population, 21.8% underwent emergency surgery, in which patients with INVE significantly required an emergency intervention than those with IPVE (29.8% vs.10.5%; *p* < 0.001).

Among those patients with IPVE, 96 patients (56.1%) represented with a previous history of combined valve surgery, 69 patients (40.4%) with isolated aortic valve replacement, 6 patients (3.5%) with isolated mitral valve replacement/resection 2 patients (1.2%) with transcatheter aortic valve implantation (TAVI).

The aortic valve was the most commonly affected valve with regurgitation of at least grade II (108; 26.3%) followed by mitral valve 78 (19.0%) and tricuspid valve 8 (1.9%).

There were 134 patients (33.1%) found with vegetation measuring 11–20 mm, followed by 63 patients (15.6%) with 5–10 mm; and 49 patients (12.1%) were found with vegetation that measured less than 5 mm. In general, large vegetation that measured 11–20 mm was commonly found on native valves than prosthetic valves (95 (40.6%) vs.39 (22.8%); < 0.001). Data of microbiological analysis showed a high incidence of infection with *Staphylococcus aureus* in 82 patients (20.0%), followed by *Enterococcus* in 61 patients (14.8%), then *Streptococcus viridans* in 43 patients (10.5%). In 113 patients (27.5%) there was no evidence of microorganisms (62 patients (25.7%) in INVE and 51 patients (30.0%) in IPVE). See Table 1.

Intraoperatively, the length of surgery was as expected significantly higher in the IPVE group (356 (299; 428) min. vs. 244 (198; 281) min.; *p* = 0.001) as well as cardiopulmonary bypass time (208 (169; 259) min. vs. 144 (111; 177) min.; *p* < 0.001) and transfusion of blood conserves (4 (0–27) vs. 2 (0–14); *p* < 0.001). Aortic valves were replaced in 305 patients (74.2%), in which 190 patients (46.2%) of them underwent valve replacement with a bioprosthetic valve and 27 patients (6.6%) underwent a mechanical valve replacement. Mitral valve surgery was carried out in 155 patients; 111 patients (27.0%) underwent bioprosthetic valve replacement, 13 (3.2%) patients underwent a mechanical valve replacement, and 31 patients (7.5%) underwent mitral valve repair with bioprosthetic annuloplasty. See Table 2.

Post-operatively, the incidence of bleeding was markedly higher in the IPVE group ((700 mL (438; 1163) vs. 500 mL (250; 1075); *p* = 0.005). Blood transfusion was markedly significant in IPVE (4 units (0–27) vs. 2 units (0–14); *p* < 0.001). Patients in the IPVE group suffered from atrioventricular bradyarrhythmia required significantly more pacemaker (17.6% vs. 7.5%: *p* = 0.002). The 30-day mortality was 17.9% (74 patients). Patients who suffered from a new onset of stroke represent 4.5% (18 patients) of the whole population without significant difference in both patients with INVE or IPVE. The 30-day mortality was 17.9% (74 patients). The mortality rate was also higher in the IPVE group (24.6% vs. 13.2%; *p* < 0.003), Table 3.

The mean follow-up was 3.1 years (0.4; 7.1). Figure 1 shows the survival curves estimated by the Kaplan–Meier method. One-year (65% vs. 79%), 3-years (58% vs. 71%) and 5-years (51% vs. 66%) survival rates were significantly lower in the IPVE group (*p* < 0.001).

A multivariate logistic regression analysis for 30-day mortality in endocarditis patients showed that patients with acute or chronic dialysis (odds ratio (OR) 2.754; *p* = 0.012), NYHA IV (OR 3.055; *p* = 0.001), neurological deficits (OR 2.976; *p* = 0.002) and abscess (OR 2.306; *p* = 0.010) were independent risk factors for mortality. Table 4.

## 4. Discussion

Despite of the huge evolution of both the role of antibiotics therapy to control and prevent the septic condition, as well as in the diagnostic and intervention tools, infective endocarditis is still considered a disease with significant morbidity and mortality [1,6,8]. The course of IE is unpredictable and in severe cases, it is difficult to be controlled, associated with a poor prognosis. Congestive heart failure followed by acute stroke is considered the most common complication of left-sided infective endocarditis, in addition to valvular incompetence.

Our analysis showed that the left-sided heart valves were commonly involved than the right ones. The aortic valve involvement comes in the first line followed by the mitral valve. Our findings went in line with several studies [8,9,10,11], where others found that the mitral valve was the most frequently affected [2,12]. IE of the right-side native valves represents <5% of all cases and it is known to be a common complication in patients with intravenous drug abusers or patients with pacemakers with intracardiac leads or those with prolonged central venous lines [13]. Isolated prosthetic endocarditis after valve surgery represents 34.6% of the study population. IPVE is associated with a high incidence of intracardiac abscess formation. Moreover, it is characterized by high recurrent rates reaching up to 15%, and poorer prognosis than in native valve IE [14,15]. Large vegetation measured more than 11 mm was found more commonly on native valves than prosthetic valves.

Microbiological analysis showed a high incidence of infection with *Staphylococcus aureus* followed by *Enterococcus* and *Streptococcus viridans*. A study by Fowler et al. found that infection with *Staphylococcus aureus* is considered an independent predictor of mortality and associated with large vegetation, high incidence of paravalvular abscesses, and low survival rate [16]. In about 27.5% of the study population, there was no evidence of microorganisms. Lamas et al. stated that up to 31% of cases with IE often represented with negative blood culture [17]. This may be contributing to the early administration of broad-spectrum antibiotics regimes before blood culture. Murdoch et al. found in his analysis that infection with *Streptococcus viridans* was associated with a decreased risk of in-hospital mortality [2].

Our results found that 18.4% suffered from preoperative stroke with a high incidence among INVE rather than IPVE. In the post-operative analysis, the new onset of stroke was relatively low (4.5%) without significant difference in both patients with INVE or IPVE. This also contributes to the early administration of antibiotics. Dickerman et al. proved the efficiency of antimicrobial therapy in a reduction in stroke rate [18]. Other studies reported an incidence of a manifested ischemic stroke reaching up to 20–35%, as well as a non-manifested neurological event reaching up to 50% [19].

In the current analysis, 21.8% of the total population underwent emergency surgery. The level of emergency depends on various factors such as hemodynamic state, degree of the septic condition, and the risk of cerebral embolism. The incidence of emergency surgery was significantly higher in patients with INVE than patients with IPVE (29.8% vs.10.5%; *p* < 0.001). The main focus of emergency surgery is for curative aims to prevent recurrent stroke and to restore the valvular function to avoid the rapid deterioration of congestive heart failure [2].

However, the ideal timing for surgical intervention in IE remains controversial. We found that the postponement of surgical therapy in patients diagnosed with IE until an occurrence of silent events—according to the guidelines and recommendation of the European Society of Cardiology (ESC) [19]—represents a point of debate. Silent events might not be well recognized or they could be interpreted otherwise. The patients might progress directly to a manifested irreversible ischemic event. Adding to that a delay to removal of intracardiac vegetation or abscess might markedly deteriorate the hemodynamic, progress to valvular and para-valvular destruction as well as the course of sepsis might be out of control regardless of the antimicrobial therapy. Various studies—in line with our study—recommend an early surgical intervention as soon as possible, regardless of stroke manifestation. A marked hemodynamic deterioration was reported due to surgical delay (after 1–2 weeks) more than the expected hazards of carrying out surgery under cardiopulmonary bypass [20,21].

The 30-day mortality rate in the current analysis reaches 17.9%, which is near to the known mortality rate of between 8–25% according to the recent ESC guidelines and other studies [6,8,10]. The risk factors for mortality were multifactorial. Our regression analysis showed that patients who required dialysis, either acute or chronic, as well as patients represented with neurological deficits and suffered from an intracardiac abscess, or those with prosthetic valve endocarditis are considered independent factors for mortality. Patients with IPVE had a significantly higher mortality rate (24.6%) when compared to INVE (13.2%). The long-term survival showed as well an acceptable result for INVE in comparison to IPVE. Our findings were confirmed by various studies regarding the risk factors of mortality and long-term survival [8,11,22]. However, other studies showed no significant differences regarding the long-term survival between both groups [10]. Moreover, various studies proved that infectious organisms could influence post-operative survival, where infections with *Staphylococcus aureus* were significantly associated with post-operative mortality when compared with non-*S. aureus* IE [8]. David TE et al. proved in an analysis that prosthetic valve endocarditis, as well as impaired ventricular function, adversely affects long-term survival, as mentioned in our study [22].

## 5. Conclusions

Patients with INVE were represented with a high incidence of preoperative embolization and neurological deficits. The aortic valve is commonly affected and patients underwent surgery frequently on an emergency basis. However, the survival rate is acceptable. Patients with IPVE were represented commonly with intracardiac abscess and characterized by high incidence of recurrence. Those patients had a higher incidence of pacemaker implantation. The mortality rate among those patients was still high.

## Figures and Tables

**Table 1:** Demographic and clinical characteristics of the study population.

	All Patients (*n* = 413)	INVE (*n* = 242, 59%)	IPVE (*n* = 171, 41%)	*p*-Value
Age, years	61.1 ± 14.9 64 (52; 73)	58.3 ± 15.5 62 (48; 69)	64.9 ± 13.2 68 (56; 75)	<0.001
Female gender	105 (25.4%)	59 (24.4%)	46 (26.9%)	0.562
EuroSCORE II	12.1 (5.2; 27.3)	6.9 (3.0; 17.0)	21.2 (12.7; 41.8)	<0.001
Chronic obstructive lung disease (COPD)	50 (12.1%)	24 (9.9%)	26 (15.2%)	0.105
Arterial hypertension	240 (58.1%)	111 (45.9%)	129 (75.4%)	<0.001
Left ventricle ejection fraction (LVEF ) (%)	55 (49; 55)	55 (50; 55)	55 (45; 55)	0.128
Drug abuse	23 (5.6%)	18 (7.4%)	5 (2.9%)	0.049
Acute dialysis preoperative	27 (6.5%)	18 (7.4%)	9 (5.3%)	0.378
Chronic dialysis preoperative	18 (4.4%)	10 (4.1%)	8 (4.7%)	0.789
NYHA IV	83 (20.2%)	56 (23.3%)	27 (15.9%)	0.064
Coronary heart disease	178 (43.2%)	92 (38.0%)	86 (50.6%)	0.011
Previous aortic valve replacement	69 (16.7%)	0 (0%)	69 (40.4%)	<0.001
Previous mitral valve replacement/resection	6 (1.5%)	0 (0%)	6 (3.5%)	<0.001
Combined valve surgery	79 (19.1%)	0 (0%)	96 (56.1%)	<0.001
Previous transcatheter aortic valve implantation (TAVI)	2 (0.5%)	0 (0%)	2 (1.2%)	<0.001
Emergency	90 (21.8%)	72 (29.8%)	18 (10.5%)	<0.001
Neurological deficits	81 (19.6%)	60 (24.8%)	21 (12.3%)	0.002
Pre-OP embolization cerebral embolization spleen other organs several organs	114 (27.6%) 58 (14.0%) 17 (4.1%) 11 (2.7%) 28 (6.8%)	86 (35.5%) 45 (18.6%) 13 (5.4%) 8 (3.3%) 20 (8.3%)	28 (16.4%) 13 (7.6%) 4 (2.3%) 3 (1.8%) 8 (4.7%)	<0.001 0.001 0.001 0.001 0.001
Pathogens				
Staphylococcus aureus Enterococcus Viridans streptococci Gram-positive streptococcus Systemic mycoses Staphylococcus epidermis other non-pathogen	82 (20.0%) 61 (14.8%) 43 (10.5%) 37 (9.0%) 6 (1.5%) 28 (6.8%) 39 (9.5%) 113 (27.5%)	54 (22.4%) 28 (11.6%) 40 (16.6%) 23 (9.5%) 2 (0.8%) 12 (5.0%) 19 (7.9%) 62 (25.7%)	28 (16.5%) 33 (19.4%) 3 (1.8%) 14 (8.2%) 4 (2.4%) 16 (9.4%) 20 (11.8%) 51 (30.0%)	
MRSA	14 (3.4%)	10 (4.1%)	4 (2.4%)	0.326
Common Affected Valve Aortic valve endocarditis Mitral valve endocarditis Tricuspid valve endocarditis Prosthetic valve endocarditis	128 (31.0%) 92 (22.3%) 7 (1.7%) 171 (41.4%)	122 (50.4%) 74 (30.6%) 5 (2.1%) 0 (0.0%)	0 (0%) 0 (0%) 0 (0%) 171 (100%)	
Common valve insufficiency (at least grade 2)				
Aortic valve Mitral valve Tricuspid valve	108 (26.3%) 78 (19.0%) 8 (1.9%)	103 (42.9%) 67 (27.9%) 6 (2.5%)	5 (2.9%) 11 (6.4%) 2 (1.2%)	
Peri-annular Abscess	113 (27.8%)	37 (15.7%)	76 (44.4%)	<0.001
Vegetation <5 mm 5–10 mm 11–20 mm >20 mm	285 (70.4%) 49 (12.1%) 63 (15.6%) 134 (33.1%) 39 (9.6%)	184 (78.6%) 29 (12.4%) 33 (14.1%) 95 (40.6%) 27 (11.5%)	101 (59.1%) 20 (11.7%) 30 (17.5%) 39 (22.8%) 12 (7.0%)	<0.001

**Table 2:** Operative data.

	All Patients (*n* = 413)	INVE (*n* = 242, 59%)	IPVE (*n* = 171, 41%)	*p*-Value
Length of surgery (min)	273 (220; 355)	244 (198; 281)	356 (299; 428)	<0.001
Cardiopulmonary bypass time (min)	166 (125; 215)	144 (111; 177)	208 (169; 259)	<0.001
Cross-clamp time (min)	116 (86; 156)	99 (77; 124)	154 (117; 181)	<0.001
Circulatory arrest (min)	0 (0–36)	0 (0–32)	0 (0–36)	<0.001
Number of packed red blood cells, unit	3 (0–27)	2 (0–14)	4 (0–27)	<0.001
Number of fresh frozen plasma, unit	0 (0–13)	0 (0–8)	0 (0–13)	<0.001
Number of platelets, unit	1 (0–6)	1 (0–6)	1 (0–6)	<0.001
Aortic valve surgery: Biological replacement Mechanical replacement Aortic root replacement	305 (74.2%) 190 (46.2%) 27 (6.6%) 80 (19.5%)	162 (66.9%) 124 (51.2%) 20 (8.3%) 15 (6.2%)	143 (84.6%) 66 (39.1%) 7 (4.1%) 65 (38.5%)	<0.001 <0.001 <0.001 <0.001
Mitral valve surgery: Biological replacement Mechanical replacement Repair surgery	155 (37.7%) 111 (27.0%) 13 (3.2%) 31 (7.5%)	114 (47.1%) 78 (32.2%) 12 (5.0%) 24 (9.9%)	41 (24.3%) 33 (19.5%) 1 (0.6%) 7 (4.1%)	<0.001 <0.001 <0.001 <0.001
Tricuspid valve surgery Biological replacement Repair surgery	15 (3.6%) 3 (0.7%) 12 (2.9%)	13 (5.4%) 2 (0.8%) 11 (4.5%)	2 (1.2%) 1 (0.6%) 1 (0.6%)	0.026 0.033 0.033

**Table 3:** Post-operative data and outcomes.

	All Patients (*n* = 413)	INVE (*n* = 242, 59%)	IPVE (*n* = 171, 41%)	*p*-Value
AKI KDIGO	115 (29.3%)	59 (25.1%)	56 (35.4%)	0.027
New–onset of Hemodialysis	61 (15.6%)	31 (13.2%)	30 (19.0%)	0.124
Hemodialysis, days	5 (3; 9)	5 (3; 8)	4 (2; 13)	0.527
24 h-drainage loss (mL)	600 (300; 1100)	500 (250; 1075)	700 (438; 1163)	0.005
Rethoracotomy due to bleeding/tamponade	50 (12.4%)	30 (12.6%)	20 (12.1%)	0.897
24 h-Number of packed red blood cells, unit,	2 (0–27)	2 (0–21)	2 (0–27)	0.334
24 h-Number of fresh frozen plasma, unit,	0 (0–29)	0 (0–18)	3 (0–29)	<0.001
24 h-Number of platelets, unit,	0 (0–8)	0 (0–5)	0 (0–8)	0.359
48 h-number of packed red blood cells, unit,	2 (0–27)	2 (0–23)	2 (0–27)	0.401
48 h-number of fresh frozen plasma, unit,	0 (0–35)	0 (0–24)	3 (0–35)	0.001
48 h-number of platelets, unit,	0 (0–9)	0 (0–6)	0 (0–9)	0.673
Ventilation time (h)	16 (9; 45)	15 (9; 46)	17 (10; 44)	0.323
Reintubation	49 (12.3%)	29 (12.1%)	20 (12.5%)	0.901
Tracheotomy	57 (14.5%)	32 (13.7%)	25 (15.8%)	0.554
Intensive care unit stay (d)	3 (1; 7)	3 (1; 7)	3 (1; 8)	0.381
Post-operative delirium	64 (16.1%)	33 (13.8%)	31 (19.7%)	0.112
Neurologic damage	27 (6.8%)	15 (6.3%)	12 (7.6%)	0.617
Cardiopulmonary resuscitation	22 (5.5%)	12 (5.0%)	10 (6.3%)	0.591
Pacemaker patient	47 (11.6%)	18 (7.5%)	29 (17.6%)	0.002
Post-operative myocardial infarction	5 (1.3%)	4 (1.7%)	1 (0.6%)	0.652
Bronchopulmonary infection	45 (11.1%)	25 (10.4%)	20 (12.0%)	0.607
Sepsis	54 (13.3%)	28 (11.7%)	26 (15.6%)	0.254
Sternal wound infection	9 (2.5%)	7 (3.1%)	2 (1.4%)	0.492
30 d-Mortality	74 (17.9%)	32 (13.2%)	42 (24.6%)	0.003
Common causes of death				
Cardiac death	10 (14.3%)	2 (6.9%)	8 (19.5%)	0.247
Cerebral death	1 (1.4%)	0 (0%)	1 (2.4%)	0.247
Sepsis	9 (12.9%)	3 (10.3%)	6 (14.6%)	0.247
Survival/follow-up time (years)	3.1 (0.4; 7.1)	3.8 (0.9; 8.2)	1.8 (0.1; 5.3)	<0.001

AKI: Acute kidney injury; KDIGO: Kidney Disease Improving Global Outcomes.

**Table 4:** Logistic regression analysis for 30-day mortality in endocarditis patients.

Predictors	Odds Ratio	95% CI	*p*-Value
Female gender	2.076	1.095–3.934	0.025
Age (years)	1.028	1.004–1.053	0.021
Dialysis (acute and chronic)	2.754	1.247–6.080	0.012
NYHA 4	3.055	1.544–6.044	0.001
Prosthetic valve endocarditis	2.162	1.085–4.311	0.028
Cardiogenic shock	3.946	1.199–12.990	0.024
Neurological deficits (TIA or stroke)	2.976	1.481–5.981	0.002
Abscess	2.306	1.220–4.361	0.010

CI: Confidence interval; TIA: Transient ischemic attack.

## Data Availability

Data is available upon requirement.
